# Luigi Maiuri: un Grande Uomo - a Great Spirit

**DOI:** 10.1038/s41419-019-1466-8

**Published:** 2019-03-01

**Authors:** Mauro Piacentini, Guido Kroemer

**Affiliations:** 1 Department of Biology, University of Rome ‘Tor Vergata’, Rome, Italy; 2 National Institute for Infectious Diseases IRCCS ‘L. Spallanzani’, Rome, Italy; 3 Equipe labellisée par la Ligue contre le cancer, Université Paris Descartes, Université Sorbonne Paris Cité, Université Paris Diderot, Université Sorbonne Université, INSERM U1138, Centre de Recherche des Cordeliers, Paris, France; 4 Metabolomics and Cell Biology Platforms, Institut Gustave Roussy, Villejuif, France; 5 Pôle de Biologie, Hôpital Européen Georges Pompidou, AP-HP, Paris, France; 6 Suzhou Institute for Systems Biology, Chinese Academy of Sciences, Suzhou, China; 7 Karolinska Institute, Department of Women’s and Children’s Health, Karolinska University Hospital, Stockholm, Sweden


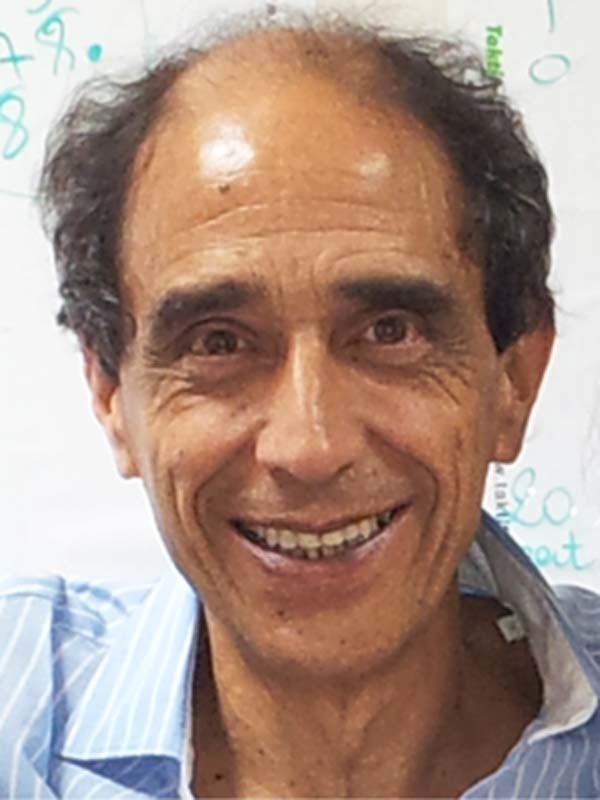


On the frontispiece of the Parisian Pantheon, the following inscription is engraved: “AUX GRANDS HOMMES, LA PATRIE RECONNAISSANTE” (“To the Great Men, the Grateful Homeland”). Logically, one of the hotels close to the Pantheon is called “Aux Grands Hommes” (always with capital letters), where Luigi stayed usually when he came to Paris. Today the inscription on the Pantheon has become “politically incorrect”, because it is not any more acceptable that humanity is only represented by men and because “homeland” has the odour of rancid patriotism. It is not any more conceivable that a country aspires at representing the entire universe as this was in the case during the French Revolution. The translation of the sense of the inscription to contemporary English might be something like “To the great spirits, the grateful universe”.

Luigi was rather small in stature and thin in complexion, nervous in his gesticulation and baroque in his expression, and he was a “Grand Homme” (in Italian “Grande Uomo”), a Great Spirit, a scientist and paediatrician with exquisite manners, irradiating an exceptional kindness, complete dedication to translational research, and an incisive intellectual curiosity.

Luigi (born in 1954, in Cosenza, Southern Italy), studied medicine in Rome, became a paediatrician in Naples, transited the University of Foggia, worked as honorary lecturer and professor at University College London and University of Southampton, and finally was affiliated to the University of Piemonte Orientale and the San Raffaele Hospital in Milan, where he was directing the European Institute for Research in Cystic Fibrosis.

Luigi’s research was always dedicated to the exploration of the molecular mechanism of human pathologies. He started his own research group relatively late, and his team was the first to identify the importance of innate immune responses and interleukin-15 for the pathogenesis of celiac disease^1^ and to develop mouse models of autoimmune thyroiditis^2^.

Luigi spent most of his carrier on cystic fibrosis, which is the most frequent monogenetic lethal disease in humans. He discovered that cystic fibrosis is linked to a defect in autophagy in respiratory epithelial cells and other cell types^3–5^. This important discovery allowed him to develop novel combination therapies involving autophagy inducers, including the combination of cysteamine and epigallocatechine gallate. Using mouse models of cystic fibrosis, he showed that both agents act on target to improve cystic fibrosis by inhibiting transglutaminase-2 and by stimulating autophagy, thus engaging in a synergistic interaction^6^. In two independent clinical phase II trials, Luigi demonstrated that the combination of cysteamine and epigallocatechine gallate is active in children bearing the most frequent pathogenic mutation in the gene coding for cystic fibrosis transmembrane conductance regulator (CFTRdel508)^7–10^. He developed and proved the concept that drugs used for the treatment of cystic fibrosis can be tested on freshly brushed nasal epithelial cells and that such in vitro test accurately predict the in vivo response in the clinics^11,12^. He also pioneered the idea that the clinical efficacy of drugs should be assessed by assessing surrogate markers, such as measuring the functionality of CFTR ex vivo^13,14^. Within the realm of cell biology, Luigi developed the concept that cystic fibrosis causes a pro-inflammatory stress response that should be targeted by modulators of proteostasis including autophagy inducers, which thus would constitute an etiological treatment^15–17^.

Recently, Luigi discovered with his dedicated team of collaborators including his wife Valeria Raia that CFTR is not only involved in the pathogenesis of cystic fibrosis, the condition in which CFTR is inactivated due to loss-of-function mutations. Indeed, Luigi found that CFTR expressed on the surface of intestinal epithelial cells can be inhibited by a specific peptide from gliadin, a gluten compound, thus explaining the pathogenesis of celiac disease. A particular non-digested peptide from gliadin binds to CFTR, thus causing its inhibition in enterocytes, resulting in autophagy inhibition, transglutaminase-2 activation, and pro-inflammatory stress^18–20^. This molecular cascade then lays the ground for stimulating an immune response against another gliadin-derived peptide fragment, resulting in a full-blown (auto-)immune response. Importantly, so called CFTR potentiators, i.e. agents that activate the chloride channel function of CFTR, can reduce the gliadin-mediated inhibition of CFTR, thus interrupting the pathogenic cascade at its apex and preventing the development of celiac disease in suitable mouse models^19,20^.

Luigi’s fertile carrier was truncated on 9 February 2019. As his memory will persist in his family, friends, alumni, colleagues, and patients, Luigi will survive as a Great Spirit in the Pantheon of Science and Medicine.
